# 2-Bromo-5-fluoro­benzaldehyde

**DOI:** 10.1107/S1600536813018783

**Published:** 2013-07-13

**Authors:** Robert E. Tureski, Joseph M. Tanski

**Affiliations:** a Department of Chemistry, Vassar College, Poughkeepsie, NY 12604, USA

## Abstract

In the title compound, C_7_H_4_BrFO, the benzaldehyde O atom is found to be *trans* to the 2-bromo substituent. In the crystal, short Br⋯F inter­actions between the bromine and fluorine substituents are observed at distances of 3.1878 (14), 3.3641 (13) and 3.3675 (14) Å. Offset face-to-face π-stacking inter­actions are also observed for both of the independent mol­ecules in the asymmetric unit running parallel to the crystallographic *b* axis, characterized by centroid–centroid distances of 3.8699 (2) and 3.8699 (2) Å.

## Related literature

For information on the synthesis of 2-bromo-5-fluoro­benzaldehyde, see: Dubost *et al.* (2011 ▶). For vibrational spectroscopic analysis and *ab initio* structure calculations on 2-bromo-5-fluoro­benzaldehyde, see: Hiremath & Sundius (2009 ▶). For the use of 2-bromo-5-fluoro­benzaldehyde in organic synthesis of biologically active compounds, see: Chen *et al.* (2013 ▶). For additional information on halogenated aromatic aldehydes in crystal structures, see: Byrn *et al.* (1993 ▶); Moorthy *et al.* (2003 ▶)*.* For information on halogen–halogen inter­actions in crystal structures, see: Pedireddi *et al.* (1994 ▶).
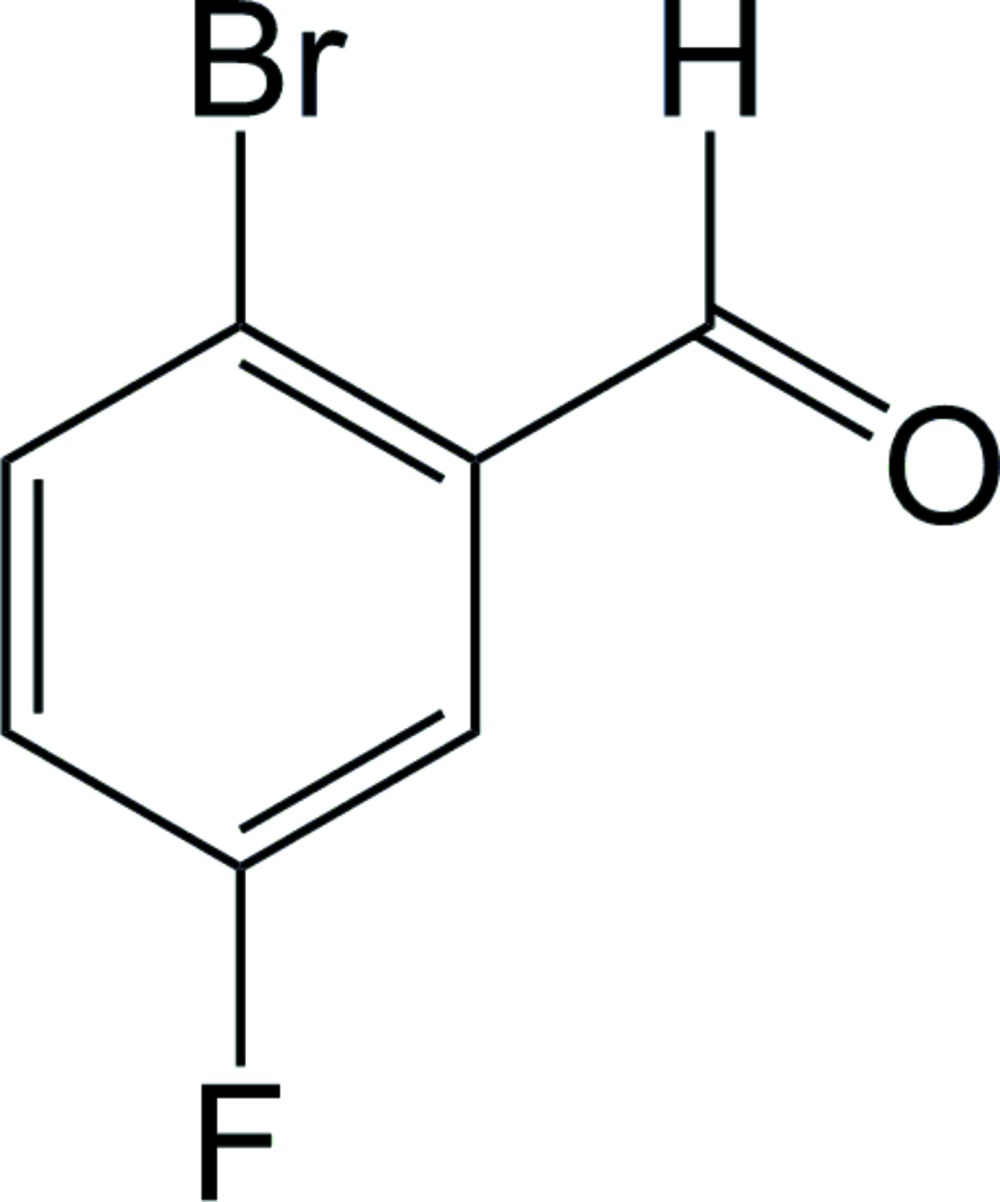


## Experimental

### 

#### Crystal data

C_7_H_4_BrFO
*M* *_r_* = 203.01
Monoclinic, 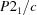

*a* = 15.3593 (6) Å
*b* = 3.8699 (2) Å
*c* = 23.4189 (9) Å
β = 106.330 (1)°
*V* = 1335.84 (10) Å^3^
*Z* = 8
Mo *K*α radiation
μ = 6.09 mm^−1^
*T* = 125 K
0.36 × 0.16 × 0.03 mm

#### Data collection

Bruker APEXII CCD diffractometer
Absorption correction: multi-scan (*SADABS*; Bruker, 2007 ▶) *T*_min_ = 0.218, *T*_max_ = 0.838
20203 measured reflections
4080 independent reflections
3468 reflections with *I* > 2σ(*I*)
*R*_int_ = 0.033

#### Refinement

*R*[*F* ^2^ > 2σ(*F* ^2^)] = 0.027
*wR*(*F* ^2^) = 0.068
*S* = 1.03
4080 reflections
181 parameters
H-atom parameters constrained
Δρ_max_ = 1.83 e Å^−3^
Δρ_min_ = −0.82 e Å^−3^

### 

Data collection: *APEX2* (Bruker, 2007 ▶); cell refinement: *SAINT* (Bruker, 2007 ▶); data reduction: *SAINT*; program(s) used to solve structure: *SHELXS97* (Sheldrick, 2008 ▶); program(s) used to refine structure: *SHELXL97* (Sheldrick, 2008 ▶); molecular graphics: *SHELXTL* (Sheldrick, 2008 ▶); software used to prepare material for publication: *SHELXTL*, *OLEX2* (Dolomanov *et al.*, 2009 ▶) and *Mercury* (Macrae *et al.*, 2006 ▶).

## Supplementary Material

Crystal structure: contains datablock(s) I, New_Global_Publ_Block. DOI: 10.1107/S1600536813018783/jj2171sup1.cif

Structure factors: contains datablock(s) I. DOI: 10.1107/S1600536813018783/jj2171Isup2.hkl

Supplementary material file. DOI: 10.1107/S1600536813018783/jj2171Isup3.cml

Additional supplementary materials:  crystallographic information; 3D view; checkCIF report

## Figures and Tables

**Fig. 1.**
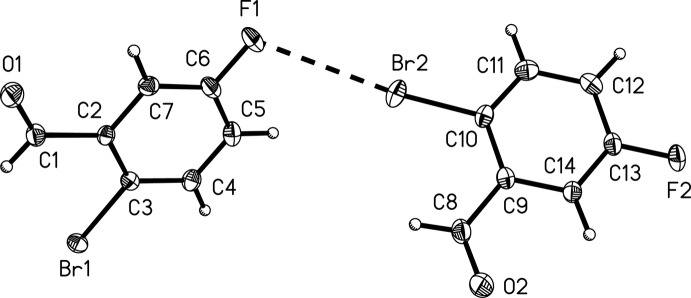
A view of the title compound with the atom numbering scheme. F1···Br2 distance 3.1878 (14) Å. Displacement ellipsoids are shown at the 50% probability level.

**Fig. 2.**
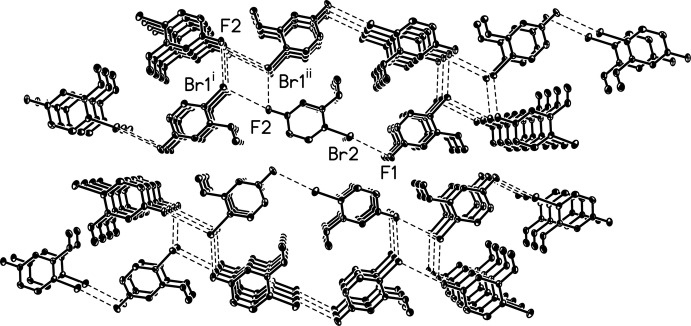
A view of the offset face-to-face π-stacking in the structure of title compound, characterized by a centroid-to-centroid distance of 3.8699 (2) Å, centroid-to-plance distance of 3.371 (2) Å, and ring-offset of 1.901 (3) Å for molecule 1 and centroid-to-centroid distance of 3.8699 (2) Å, centroid-to-plance distance of 3.431 (2) Å, and ring-offset of 1.790 (3) Å for molecule 2. F1···Br2 distance 3.1878 (14) Å, F2···Br1i distance 3.3641 (13), and F2···Br1ii distance 3.3675 (14) Å. Displacement ellipsoids are shown at the 50% probability level. Symmetry codes: (i) x + 1, y + 1, z; (ii) -x + 1, y + 1/2, -z + 1/2.

## supplementary crystallographic information

### Comment

*Ab initio* electronic structure calculations have predicted that the lower energy conformer of 2-bromo-5-fluorobenzaldehyde is the one in which the benzaldehyde oxygen is *trans* to the 2-bromo substituent (Hiremath & Sundius, 2009). The crystal structure of 2-bromo-5-fluorobenzaldehyde reported here is consistent with this finding. The compound may be synthesized by selective *ortho*-bromination of the appropriate benzaldoxime (Dubost *et al.*, 2011). The substance has found laboratory application in the synthesis of quinazolinones which are known to show antitumor activity (Chen *et al.*, 2013). Crystal structures of halogenated aromatic aldehydes have been previously reported (Moorthy *et al.*, 2003), and they are also known in clathrates (Byrn *et al.*,1993).

The titular compound crystallizes with two molecules of 2-bromo-5-fluorobenzaldehyde in the asymmetric unit (Fig. 1). The benzaldehyde oxygen is *trans* to the 2-bromo substituent, with O1—C1—C2—C3 and O2—C8—C9—C10 torsional angles of 174.3 (2)° and 170.2 (2)°, respectively. Several short intermolecuar halogen-halogen interactions can be seen in the structure between fluorine and bromine atoms. Within the asymmetric unit, the F1···Br2 distance of 3.1878 (14) Å is significantly shorter than the sum of the van der Waals radii of bromine and fluorine, 3.40 Å (Pedireddi *et al.*, 1994). Somewhat longer, the F2···Br1^i^ distance 3.3641 (13) and F2···Br1^ii^ distance 3.3675 (14) Å are about the same as the sum of the van der Waals radii of fluorine and bromine (Fig 2.)

The aromatic compound packs in the solid state with an offset face-to-face π-stacking motif parallel to the crystallographic *b*-axis (Fig 2.). Each independent molecule in the asymmetric unit π-stacks with itself. This π-stacking motif is characterized by a centroid-to-centroid distance of 3.8699 (2) Å, centroid-to-plance distance of 3.371 (2) Å, and ring-offset of 1.901 (3) Å for molecule 1 and centroid-to-centroid distance of 3.8699 (2) Å, centroid-to-plance distance of 3.431 (2) Å, and ring-offset of 1.790 (3) Å for molecule 2.

### Experimental

2-bromo-5-fluorobenzaldehyde was purchased from Aldrich Chemical Company, USA, and was recrystallized from chloroform.

### Refinement

All non-hydrogen atoms were refined anisotropically. Hydrogen atoms on carbon were included in calculated positions and refined using a riding model at C–H = 0.95 Å and *U*_iso_(H) = 1.2 × *U*_eq_(C) of the aryl C-atoms. The extinction parameter (EXTI) refined to zero and was removed from the refinement.

### Crystal data

**Table:**

C_7_H_4_BrFO	*F*(000) = 784
*M**_r_* = 203.01	*D*_x_ = 2.019 Mg m^−^^3^
Monoclinic, *P*2_1_/*c*	Mo *K*α radiation, λ = 0.71073 Å
Hall symbol: -P 2ybc	Cell parameters from 9913 reflections
*a* = 15.3593 (6) Å	θ = 2.6–30.5°
*b* = 3.8699 (2) Å	µ = 6.09 mm^−^^1^
*c* = 23.4189 (9) Å	*T* = 125 K
β = 106.330 (1)°	Plate, colourless
*V* = 1335.84 (10) Å^3^	0.36 × 0.16 × 0.03 mm
*Z* = 8	

### Data collection

**Table:**

Bruker APEXII CCD diffractometer	4080 independent reflections
Radiation source: fine-focus sealed tube	3468 reflections with *I* > 2σ(*I*)
Graphite monochromator	*R*_int_ = 0.033
φ and ω scans	θ_max_ = 30.5°, θ_min_ = 1.8°
Absorption correction: multi-scan (*SADABS*; Bruker, 2007)	*h* = −21→21
*T*_min_ = 0.218, *T*_max_ = 0.838	*k* = −5→5
20203 measured reflections	*l* = −33→33

### Refinement

**Table:**

Refinement on *F*^2^	Primary atom site location: structure-invariant direct methods
Least-squares matrix: full	Secondary atom site location: difference Fourier map
*R*[*F*^2^ > 2σ(*F*^2^)] = 0.027	Hydrogen site location: inferred from neighbouring sites
*wR*(*F*^2^) = 0.068	H-atom parameters constrained
*S* = 1.03	*w* = 1/[σ^2^(*F*_o_^2^) + (0.0289*P*)^2^ + 1.4137*P*] where *P* = (*F*_o_^2^ + 2*F*_c_^2^)/3
4080 reflections	(Δ/σ)_max_ = 0.001
181 parameters	Δρ_max_ = 1.83 e Å^−^^3^
0 restraints	Δρ_min_ = −0.82 e Å^−^^3^

### Special details

**Table:**

Geometry. All e.s.d.'s (except the e.s.d. in the dihedral angle between two l.s. planes) are estimated using the full covariance matrix. The cell e.s.d.'s are taken into account individually in the estimation of e.s.d.'s in distances, angles and torsion angles; correlations between e.s.d.'s in cell parameters are only used when they are defined by crystal symmetry. An approximate (isotropic) treatment of cell e.s.d.'s is used for estimating e.s.d.'s involving l.s. planes.
Refinement. Refinement of *F*^2^ against ALL reflections. The weighted *R*-factor *wR* and goodness of fit *S* are based on *F*^2^, conventional *R*-factors *R* are based on *F*, with *F* set to zero for negative *F*^2^. The threshold expression of *F*^2^ > σ(*F*^2^) is used only for calculating *R*-factors(gt) *etc*. and is not relevant to the choice of reflections for refinement. *R*-factors based on *F*^2^ are statistically about twice as large as those based on *F*, and *R*- factors based on ALL data will be even larger.

### Fractional atomic coordinates and isotropic or equivalent isotropic displacement parameters (Å^2^)

**Table:**

	*x*	*y*	*z*	*U*_iso_*/*U*_eq_	
Br1	0.080111 (13)	0.22202 (5)	0.216581 (8)	0.01891 (6)	
Br2	0.462736 (14)	0.76345 (5)	0.083534 (10)	0.02508 (6)	
F1	0.25127 (9)	0.6309 (4)	0.02624 (6)	0.0291 (3)	
F2	0.85470 (8)	1.1648 (4)	0.15460 (6)	0.0280 (3)	
O1	−0.05661 (10)	0.8333 (4)	0.05688 (7)	0.0262 (3)	
O2	0.60148 (11)	1.3284 (5)	0.24807 (7)	0.0319 (4)	
C1	−0.01416 (13)	0.6545 (5)	0.09806 (9)	0.0192 (4)	
H1A	−0.0436	0.5795	0.1265	0.023*	
C2	0.08121 (12)	0.5479 (5)	0.10601 (8)	0.0154 (3)	
C3	0.13221 (13)	0.3679 (5)	0.15590 (8)	0.0156 (3)	
C4	0.22246 (13)	0.2828 (5)	0.16300 (9)	0.0194 (4)	
H4A	0.2564	0.1646	0.1977	0.023*	
C5	0.26256 (14)	0.3727 (5)	0.11871 (9)	0.0219 (4)	
H5A	0.3241	0.3164	0.1225	0.026*	
C6	0.21117 (14)	0.5449 (5)	0.06927 (9)	0.0206 (4)	
C7	0.12254 (13)	0.6377 (5)	0.06165 (8)	0.0181 (4)	
H7A	0.0898	0.7601	0.0271	0.022*	
C8	0.57143 (14)	1.1450 (6)	0.20481 (9)	0.0226 (4)	
H8A	0.5118	1.0545	0.1978	0.027*	
C9	0.62435 (13)	1.0572 (5)	0.16259 (8)	0.0166 (3)	
C10	0.58708 (13)	0.8909 (5)	0.10820 (9)	0.0176 (3)	
C11	0.63924 (15)	0.8169 (5)	0.06990 (9)	0.0217 (4)	
H11A	0.6125	0.7055	0.0330	0.026*	
C12	0.73049 (14)	0.9061 (5)	0.08565 (9)	0.0218 (4)	
H12A	0.7673	0.8545	0.0602	0.026*	
C13	0.76605 (13)	1.0719 (5)	0.13929 (9)	0.0191 (4)	
C14	0.71611 (13)	1.1487 (5)	0.17800 (8)	0.0181 (3)	
H14A	0.7435	1.2621	0.2146	0.022*	

### Atomic displacement parameters (Å^2^)

**Table:**

	*U*^11^	*U*^22^	*U*^33^	*U*^12^	*U*^13^	*U*^23^
Br1	0.02187 (10)	0.01889 (9)	0.01725 (9)	−0.00061 (7)	0.00756 (7)	0.00286 (7)
Br2	0.01723 (10)	0.02099 (11)	0.03344 (12)	−0.00366 (7)	0.00124 (8)	−0.00262 (8)
F1	0.0273 (7)	0.0388 (7)	0.0277 (6)	−0.0023 (6)	0.0182 (5)	0.0033 (6)
F2	0.0148 (6)	0.0395 (7)	0.0314 (7)	−0.0053 (5)	0.0095 (5)	0.0010 (6)
O1	0.0215 (7)	0.0328 (8)	0.0237 (7)	0.0061 (6)	0.0051 (6)	0.0075 (6)
O2	0.0253 (8)	0.0419 (9)	0.0317 (9)	−0.0034 (7)	0.0130 (7)	−0.0137 (7)
C1	0.0164 (8)	0.0208 (9)	0.0212 (9)	−0.0006 (7)	0.0064 (7)	0.0012 (7)
C2	0.0140 (8)	0.0145 (8)	0.0174 (8)	−0.0019 (6)	0.0038 (7)	−0.0007 (6)
C3	0.0175 (8)	0.0134 (8)	0.0168 (8)	−0.0017 (6)	0.0062 (7)	−0.0009 (6)
C4	0.0171 (9)	0.0176 (9)	0.0224 (9)	0.0011 (7)	0.0039 (7)	0.0010 (7)
C5	0.0160 (9)	0.0207 (9)	0.0298 (10)	0.0002 (7)	0.0080 (8)	−0.0013 (8)
C6	0.0222 (10)	0.0216 (9)	0.0219 (9)	−0.0034 (7)	0.0126 (8)	−0.0017 (7)
C7	0.0196 (9)	0.0179 (8)	0.0164 (8)	−0.0032 (7)	0.0044 (7)	−0.0011 (7)
C8	0.0164 (9)	0.0259 (10)	0.0267 (10)	−0.0005 (8)	0.0081 (8)	−0.0012 (8)
C9	0.0152 (8)	0.0151 (8)	0.0206 (9)	0.0006 (6)	0.0066 (7)	0.0014 (7)
C10	0.0145 (8)	0.0144 (8)	0.0221 (9)	0.0000 (6)	0.0023 (7)	0.0016 (7)
C11	0.0260 (10)	0.0190 (9)	0.0190 (9)	0.0016 (8)	0.0047 (8)	0.0007 (7)
C12	0.0250 (10)	0.0224 (9)	0.0212 (9)	0.0026 (8)	0.0115 (8)	0.0024 (7)
C13	0.0143 (9)	0.0214 (9)	0.0221 (9)	−0.0001 (7)	0.0061 (7)	0.0038 (7)
C14	0.0158 (8)	0.0187 (8)	0.0196 (9)	−0.0017 (7)	0.0048 (7)	0.0007 (7)

### Geometric parameters (Å, º)

**Table:**

Br1—C3	1.9021 (18)	C5—C6	1.376 (3)
Br2—C10	1.8985 (19)	C5—H5A	0.9500
Br2—F1	3.1878 (14)	C6—C7	1.370 (3)
F1—C6	1.362 (2)	C7—H7A	0.9500
F2—C13	1.355 (2)	C8—C9	1.485 (3)
F2—Br1^i^	3.3641 (13)	C8—H8A	0.9500
F2—Br1^ii^	3.3675 (14)	C9—C10	1.398 (3)
O1—C1	1.217 (3)	C9—C14	1.399 (3)
O2—C8	1.217 (3)	C10—C11	1.390 (3)
C1—C2	1.482 (3)	C11—C12	1.389 (3)
C1—H1A	0.9500	C11—H11A	0.9500
C2—C3	1.396 (3)	C12—C13	1.379 (3)
C2—C7	1.405 (3)	C12—H12A	0.9500
C3—C4	1.389 (3)	C13—C14	1.374 (3)
C4—C5	1.391 (3)	C14—H14A	0.9500
C4—H4A	0.9500		
			
C10—Br2—F1	171.36 (6)	C6—C7—H7A	120.7
C6—F1—Br2	110.32 (11)	C2—C7—H7A	120.7
C13—F2—Br1^i^	165.26 (12)	O2—C8—C9	122.51 (19)
C13—F2—Br1^ii^	97.22 (10)	O2—C8—H8A	118.7
Br1^i^—F2—Br1^ii^	68.67 (3)	C9—C8—H8A	118.7
O1—C1—C2	123.25 (18)	C10—C9—C14	118.44 (17)
O1—C1—H1A	118.4	C10—C9—C8	123.47 (17)
C2—C1—H1A	118.4	C14—C9—C8	118.09 (17)
C3—C2—C7	118.59 (17)	C11—C10—C9	121.34 (18)
C3—C2—C1	123.09 (16)	C11—C10—Br2	117.59 (15)
C7—C2—C1	118.31 (17)	C9—C10—Br2	121.06 (14)
C4—C3—C2	121.66 (17)	C12—C11—C10	119.94 (19)
C4—C3—Br1	117.11 (14)	C12—C11—H11A	120.0
C2—C3—Br1	121.23 (14)	C10—C11—H11A	120.0
C3—C4—C5	119.19 (18)	C13—C12—C11	117.98 (18)
C3—C4—H4A	120.4	C13—C12—H12A	121.0
C5—C4—H4A	120.4	C11—C12—H12A	121.0
C6—C5—C4	118.62 (18)	F2—C13—C14	118.27 (18)
C6—C5—H5A	120.7	F2—C13—C12	118.38 (17)
C4—C5—H5A	120.7	C14—C13—C12	123.35 (18)
F1—C6—C7	118.63 (18)	C13—C14—C9	118.94 (18)
F1—C6—C5	117.97 (18)	C13—C14—H14A	120.5
C7—C6—C5	123.39 (18)	C9—C14—H14A	120.5
C6—C7—C2	118.54 (18)		
			
O1—C1—C2—C3	174.3 (2)	O2—C8—C9—C14	9.3 (3)
O1—C1—C2—C7	−4.5 (3)	C14—C9—C10—C11	−0.1 (3)
C7—C2—C3—C4	1.3 (3)	C8—C9—C10—C11	179.40 (19)
C1—C2—C3—C4	−177.61 (18)	C14—C9—C10—Br2	−179.19 (14)
C7—C2—C3—Br1	−177.71 (14)	C8—C9—C10—Br2	0.3 (3)
C1—C2—C3—Br1	3.4 (3)	C9—C10—C11—C12	0.5 (3)
C2—C3—C4—C5	−1.4 (3)	Br2—C10—C11—C12	179.70 (15)
Br1—C3—C4—C5	177.64 (15)	C10—C11—C12—C13	−0.9 (3)
C3—C4—C5—C6	0.3 (3)	Br1^i^—F2—C13—C14	74.6 (5)
Br2—F1—C6—C7	148.75 (15)	Br1^ii^—F2—C13—C14	58.25 (18)
Br2—F1—C6—C5	−30.4 (2)	Br1^i^—F2—C13—C12	−105.5 (4)
C4—C5—C6—F1	−179.91 (18)	Br1^ii^—F2—C13—C12	−121.91 (16)
C4—C5—C6—C7	1.0 (3)	C11—C12—C13—F2	−178.98 (18)
F1—C6—C7—C2	179.81 (17)	C11—C12—C13—C14	0.8 (3)
C5—C6—C7—C2	−1.1 (3)	F2—C13—C14—C9	179.45 (17)
C3—C2—C7—C6	0.0 (3)	C12—C13—C14—C9	−0.4 (3)
C1—C2—C7—C6	178.90 (18)	C10—C9—C14—C13	0.0 (3)
O2—C8—C9—C10	−170.2 (2)	C8—C9—C14—C13	−179.52 (18)

Symmetry codes: (i) *x*+1, *y*+1, *z*; (ii) −*x*+1, *y*+1/2, −*z*+1/2.
